# LncRNA-UCA1 enhances MMP-13 expression by inhibiting miR-204-5p in human chondrocytes

**DOI:** 10.18632/oncotarget.20108

**Published:** 2017-08-10

**Authors:** Guodong Wang, Xianmin Bu, Yuanmin Zhang, Xiaowei Zhao, Ying Kong, Longfei Ma, Shuaishuai Niu, Bin Wu, Chunyang Meng

**Affiliations:** ^1^ Department of Orthopaedics, Affiliated Hospital of Jining Medical University, Jining, Shandong, China; ^2^ Department of Pathology, Shandong Jining No.1 People's Hospital, Jining, Shandong, China

**Keywords:** osteoarthritis, long noncoding RNAs, UCA1, miR-204-5p, MMP-13

## Abstract

Osteoarthritis (OA) is a common degenerative disease characterized by degeneration of articular cartilage. Increasing studies showed that long noncoding RNAs (lncRNAs) play important roles in the cartilage damage. However, little is known about the role of UCA1 in the osteoarthritis. The expression level of UCA1 was upregulated in the OA cartilage. Overexpression of UCA1 suppressed the miR-204-5p expression in the chondrocytes. The expression of miR-204-5p was downregulated in the OA cartilage. Moreover, the expression of miR-204-5p was negatively correlated with the UCA1 expression in the OA cartilage. Elevated expression of UCA1 promoted the chondrocytes cell proliferation and overexpression of miR-204-5p suppressed chondrocytes cell proliferation. In addition, overexpression of UCA1 decreased the expression of the type II collagen and type IV collagen expression in the chondrocytes. Elevated expression of miR-204-5p promoted the type II collagen and type IV collagen expression in the chondrocytes. We idetified MMP-13 was a direct target gene of miR-204-5p in the chondrocytes. Overexpression of UCA1 enhanced the MMP-13 expression in the chondrocytes. Elevated expression of UCA1 regulated the chondrocytes cell proliferation and collagen expression through inhibiting the miR-204-5p expression.These results suggested that UCA1 played as an important regulator of survival and matrix synthesis of chondrocytes partly through suppressing the miR-204-5p expression.

## INTRODUCTION

Osteoarthritis (OA) is a degenerative disease of the joints and is regarded by tenderness, pain, crepitus, limited movement, which is the most prevalent cause of mobility-associated disability [1–4]. The pathogenesis of OA is multifactorial and involves the interaction of several factors [5, 6]. The etiology of OA is also complex such as failure of nutrient supply, genetic predisposition, trauma and abnormal mechanical loading [7–11].

Long noncoding RNAs (lncRNAs) are a group of the noncoding RNA family and are longer than 200 nucleotides [12–15]. Accumulating evidences demonstrated that lncRNAs are involved in several cell biological processes including cell growth, differentiation, invasion, cell cycle progression, migration and apoptosis [16–20]. Additionally, lncRNAs misexpression was found in several tumors such as retinoblastoma, gastric cancer, endometrial carcinoma, breast cancer, and colorectal cancer [15, 21–25]. Recent studies have showed that lncRNAs play a crucial role in the development of arthritis and the joint homeostasis maintenance [26]. Su et al. [26]. showed that maternally expressed gene 3 (MEG3) was downregulated in OA patients compared to normal cartilage samples. Human urothelial carcinoma associated 1 (UCA1) was originally found to be upregulated in bladder tumor and was demonstrated to serve as an important regulator of cell proliferation, invasion and migration [27–30]. UCA1 was found to be upregulated in tongue squamous cell carcinomas, gastric cancer, breast cancer and bladder cancer [28, 31–34]. However, the functional role of UCA1 in OA development is still not documented.

In this study, we sought to the expression of UCA1 in the OA cartilage and normal cartilage. We showed that the expression level of UCA1 was upregulated in the OA cartilage. Overexpression of UCA1 suppressed the miR-204-5p expression and enhanced the MMP-13 expression in the chondrocytes.

## RESULTS

### The expression of UCA1 was upregulated in the OA cartilage

**Figure 1:**
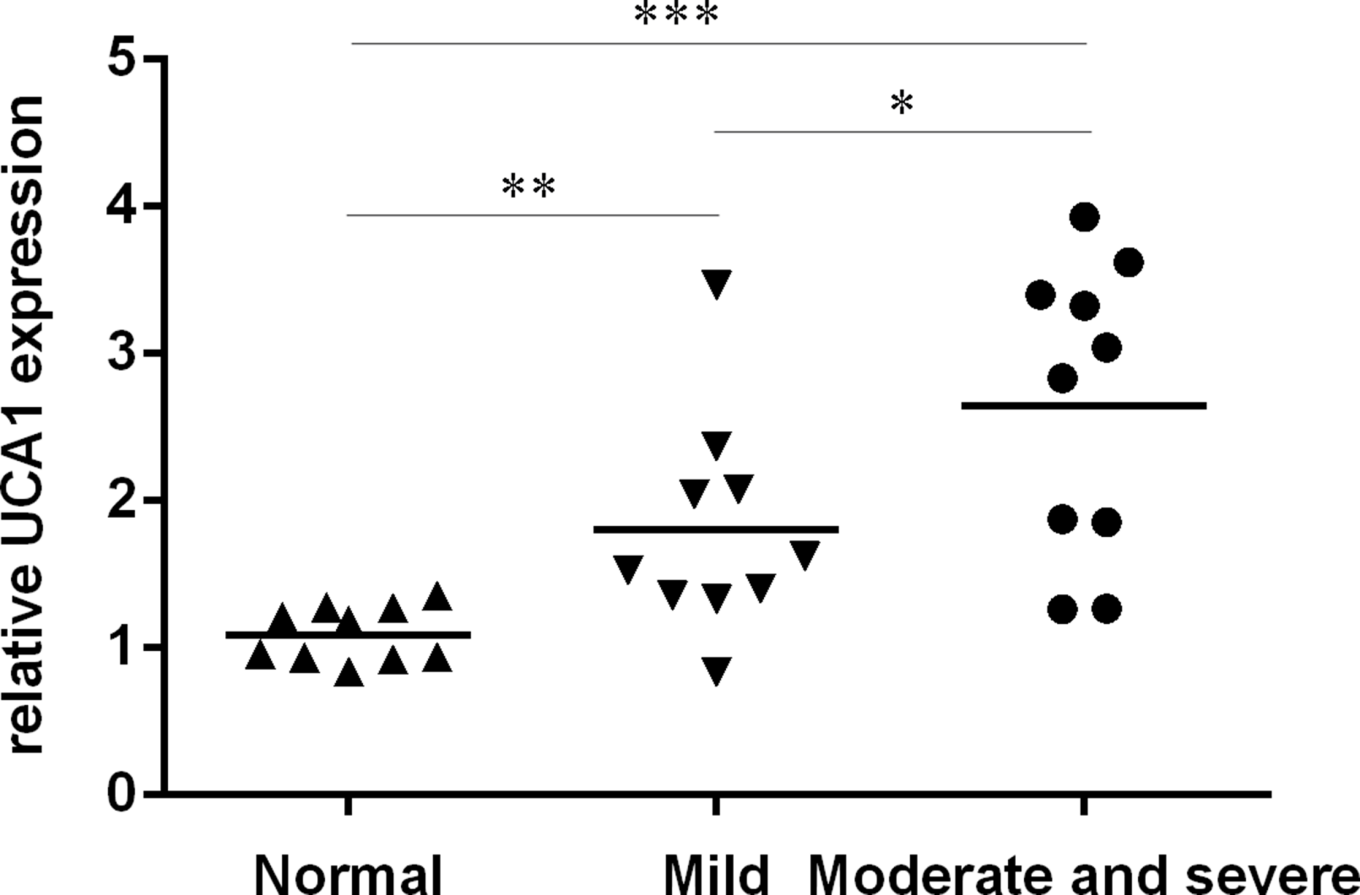
The expression of UCA1 was upregulated in the OA cartilage The expression of UCA1 in the OA cartilage and normal cartilage was determined by qRT-PCR. *p < 0.05, **p < 0.01 and ***p < 0.001.

We firstly determined the expression of UCA1 in the OA cartilage and normal cartilage. As shown in the Figure 1, the expression level of UCA1 was highest in the moderate and severe group compared to in the normal cartilage and mild OA cartilage. The data indicated that the expression level of UCA1 was upregulated in the OA cartilage.

### The expression of miR-204-5p was downregulated in the OA cartilage

**Figure 2:**
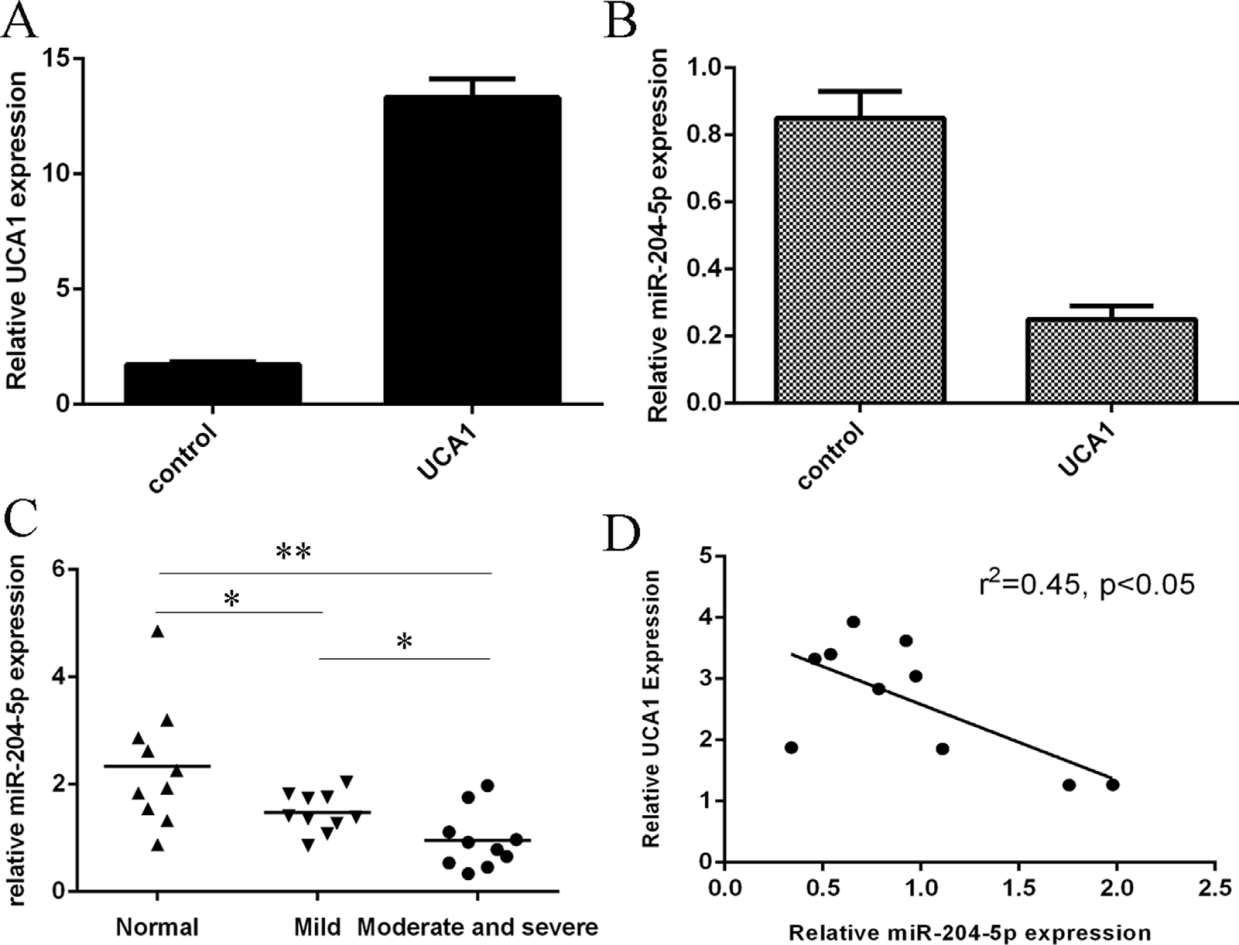
The expression of miR-204-5p was downregulated in the OA cartilage (**A**) The expression of UCA1 was measured in the chondrocytes after treated with pcDNA-UCA1. (**B**) Overexpression of UCA1 suppressed the miR-204-5p expression in the chondrocytes. (**C**) The expression level of miR-204-5p in the normal cartilage and OA cartilage was measured by qRT-PCR. (**D**) The expression of miR-204-5p was negatively correlated with the UCA1 expression in the OA cartilage. *p < 0.05 and **p < 0.01.

The expression of UCA1 was significantly upregulated in the chondrocytes after treated with pcDNA-UCA1 (Figure 2A). Overexpression of UCA1 suppressed the miR-204-5p expression in the chondrocytes (Figure 2B). We then determined the expression of miR-204-5p in the OA cartilage and normal cartilage. As shown in the Figure 2C, the expression level of miR-204-5p was lowest in the moderate and severe group compared to in the normal cartilage and mild OA cartilage. The data indicated that the expression level of miR-204-5p was downregulated in the OA cartilage. Moreover, the expression of miR-204-5p was negatively correlated with the UCA1 expression in the OA cartilage (Figure 2D).

### miR-204-5p suppressed the MMP-13 expression in the chondrocytes

**Figure 3:**
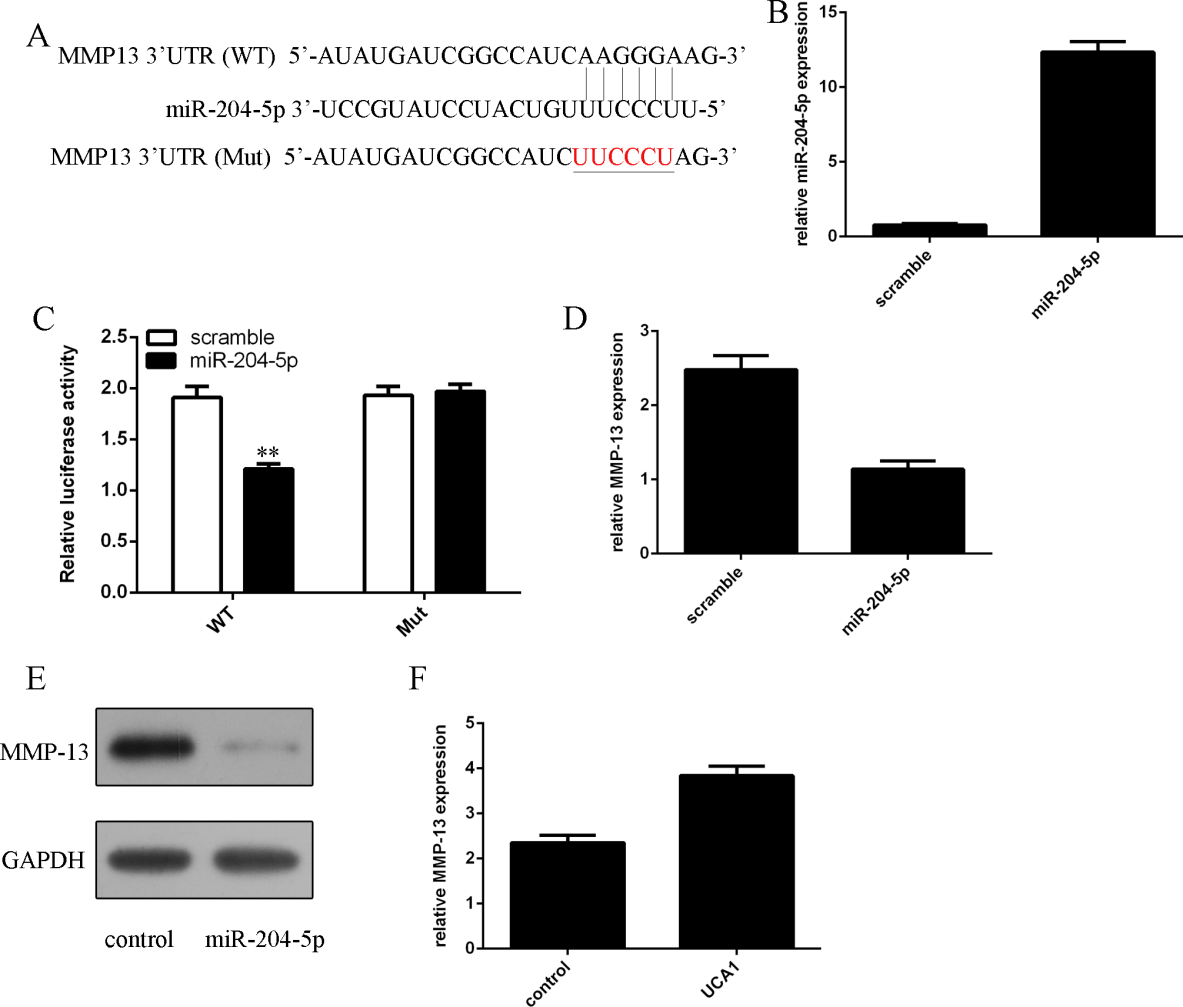
miR-204-5p suppressed the MMP-13 expression in the chondrocytes (**A**) There is a potential miR-204-5p binding sequence in the 3’UTR of MMP-13 through by using TargetScanHuman miRNA target prediction software. (**B**) The expression of miR-204-5p in the chondrocytes was measured by qRT-PCR. (**C**) miR-204-5p decreased the luciferase activity of MMP-13-WT plasmid, but not of MMP-13-Mut, manifesting that MMP-13 was one of miR-204-5p direct targets. (**D**) Overexpression of miR-204-5p suppressed MMP-13 expression in the chondrocytes. (**E**) The protein expression of MMP-13 in the chondrocytes was analyzed by western blot. (**F**) Overexpression of UCA1 promoted the MMP-13 expression in the chondrocytes. **p < 0.01.

We identified a potential miR-204-5p binding sequence in the 3′UTR of MMP-13 through using TargetScanHuman miRNA target prediction software (Figure 3A). The expression of miR-204-5p was significantly upregulated in the chondrocytes after treated with miR-204-5p mimic (Figure 3B). As shown in the Figure 3C, miR-204-5p decreased the luciferase activity of MMP-13-WT plasmid, but not of MMP-13-Mut, manifesting that MMP-13 was one of miR-204-5p direct targets. Overexpression of miR-204-5p suppressed MMP-13 expression in the chondrocytes (Figure 3D). Elevated expression of miR-204-5p also inhibited the protein expression of MMP-13 in the chondrocytes (Figure 3E). Overexpression of UCA1 promoted the MMP-13 expression in the chondrocytes (Figure 3F).

### Elevated expression of UCA1 promoted the chondrocytes cell proliferation and miR-204-5p suppressed chondrocytes cell proliferation

**Figure 4:**
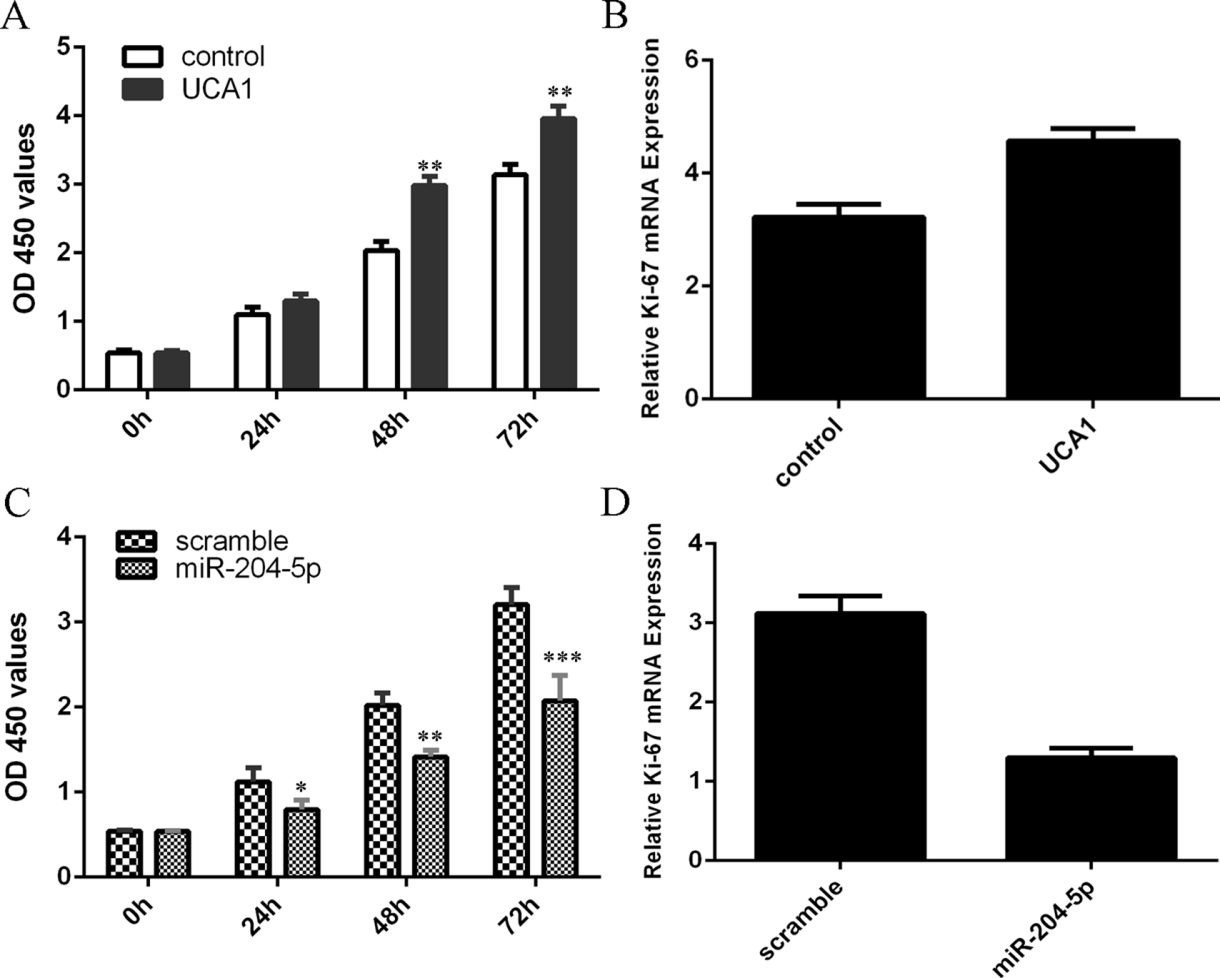
Elevated expression of UCA1 promoted the chondrocytes cell proliferation and miR-204-5p suppressed chondrocytes cell proliferation Overexpression of UCA1 increased the chondrocytes cell proliferation (**A**). Elevated expression of UCA1 promoted the ki-67 expression in the chondrocytes (**B**). Elevated expression of miR-204-5p suppressed the chondrocytes cell proliferation (**C**). Ecoptic expression of miR-204-5p inhibited the ki-67 expression in the chondrocytes (**D**). *p < 0.05, **p < 0.01 and ***p < 0.001.

Overexpression of UCA1 increased the chondrocytes cell proliferation (Figure 4A). Elevated expression of UCA1 promoted the ki-67 expression in the chondrocytes (Figure 4B). Elevated expression of miR-204-5p suppressed the chondrocytes cell proliferation (Figure 4C). Ecoptic expression of miR-204-5p inhibited the ki-67 expression in the chondrocytes (Figure 4D).

### Overexpression of UCA1 decreased the expression of the type II collagen and type IV collagen

**Figure 5:**
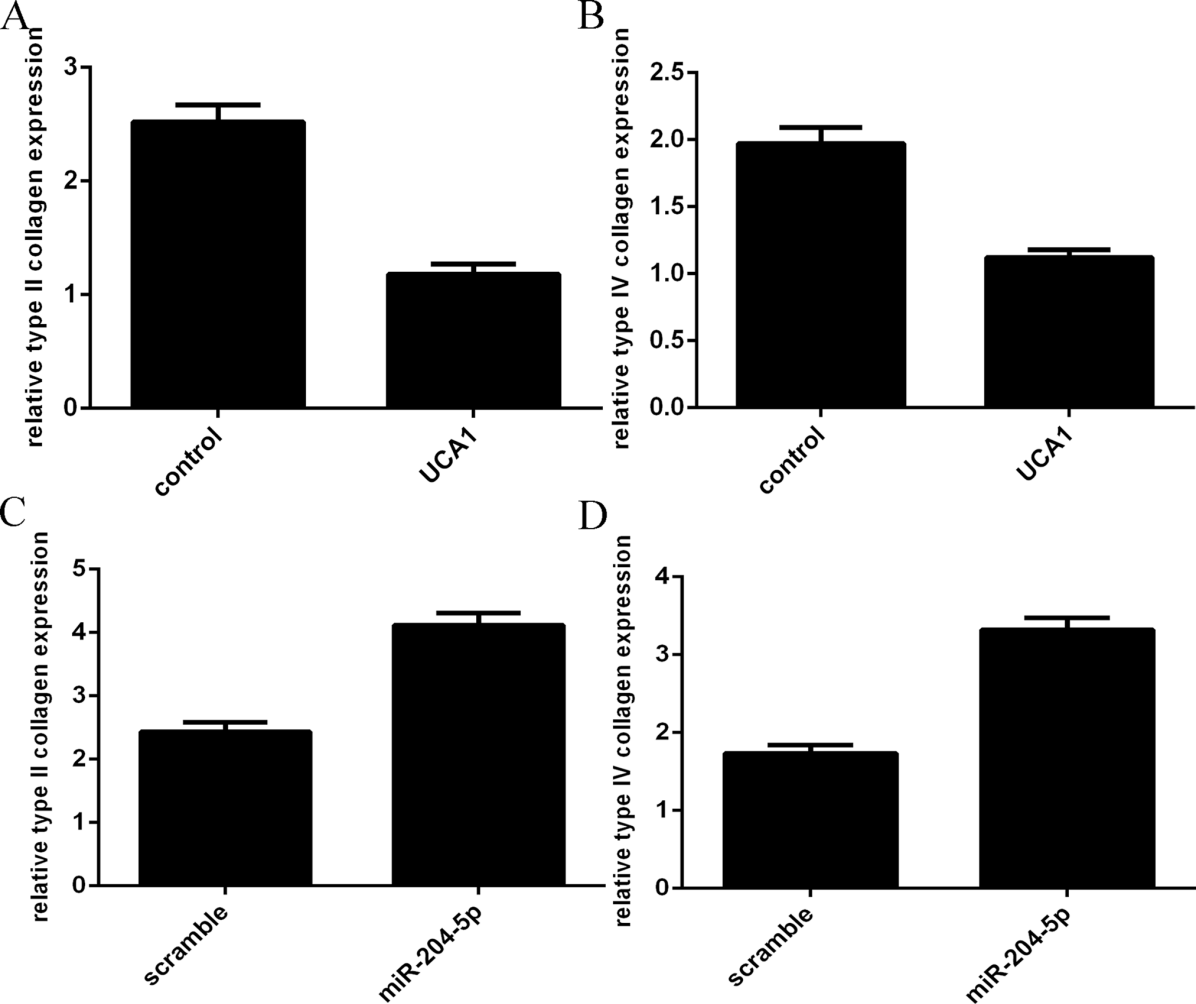
Overexpression of UCA1 decreased the expression of the type II collagen and type IV collagen (**A**) Overexpression of UCA1 suppressed the type II collagen expression in the chondrocytes. (**B**) The expression of type IV collagen in the chondrocytes was measured by qRT-PCR. (**C**) Elevated expression of miR-204-5p promoted the type II collagen expression. (**D**) The expression of type IV collagen in the chondrocytes was measured by qRT-PCR.

Overexpression of UCA1 suppressed the type II collagen (Figure 5A) and type IV collagen (Figure 5B) expression in the chondrocytes. In addition, elevated expression of miR-204-5p promoted the type II collagen (Figure 5C) and type IV collagen (Figure 5D) expression in the chondrocytes.

### Elevated expression of UCA1 regulated the chondrocytes cell proliferation and collagen expression through inhibiting the miR-204-5p expression

**Figure 6:**
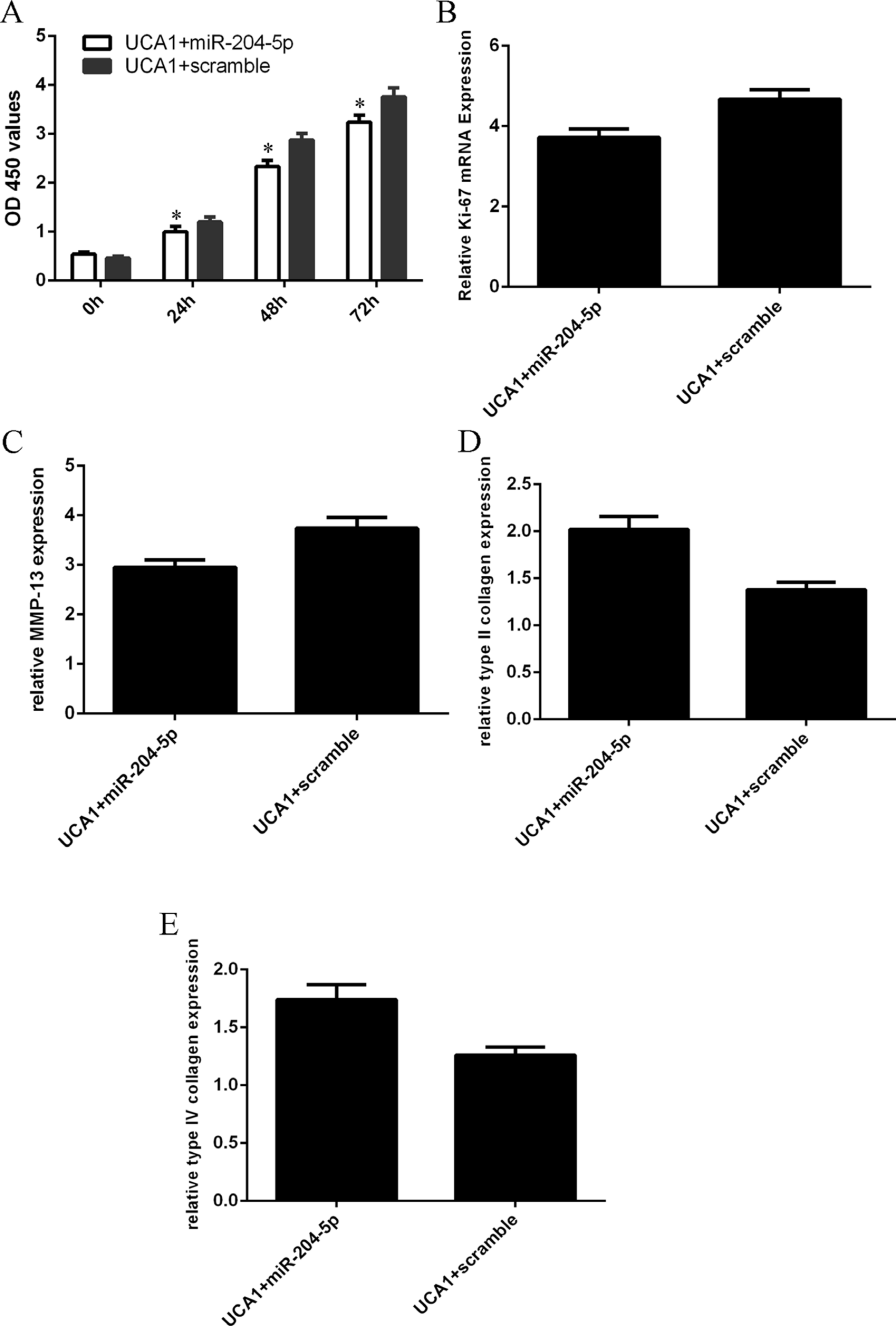
Elevated expression of UCA1 regulated the chondrocytes cell proliferation and collagen expression through inhibiting the miR-204-5p expression (**A**) Elevated expression of miR-204-5p inhibited chondrocytes cell proliferation, reversing UCA1-induced chondrocytes cell proliferation. (**B**) Overexpression of miR-204-5p also inhibited the ki-67 expression in the UCA1 overexpressing-chondrocytes. (**C**) Elevated expression of miR-204-5p suppressed the MMP-13 expression in the UCA1 overexpressing-chondrocytes. (**D**) Ecoptic expression of miR-204-5p enhanced the type II collagen expression in the UCA1 overexpressing-chondrocytes. (**E**) Ecoptic expression of miR-204-5p enhanced the type IV collagenexpression in the UCA1 overexpressing-chondrocytes. *p < 0.05.

We restored miR-204-5p expression in the UCA1 overexpressing-chondrocytes to study whether miR-204-5p was involved in the function of UCA1 in chondrocytes. Elevated expression of miR-204-5p inhibited chondrocytes cell proliferation, reversing UCA1-induced chondrocytes cell proliferation (Figure 6A). Overexpression of miR-204-5p also inhibited the ki-67 expression in the UCA1 overexpressing-chondrocytes (Figure 6B). Moreover, elevated expression of miR-204-5p suppressed the MMP-13 expression in the UCA1 overexpressing-chondrocytes (Figure 6C). Ecoptic expression of miR-204-5p enhanced the type II collagen (Figure 6D) and type IV collagen (Figure 6E) expression in the UCA1 overexpressing-chondrocytes.

## DISCUSSION

In our study, we found that the expression level of UCA1 was upregulated in the OA cartilage. Overexpression of UCA1 suppressed the miR-204-5p expression in the chondrocytes. The expression of miR-204-5p was downregulated in the OA cartilage. Moreover, the expression of miR-204-5p was negatively correlated with the UCA1 expression in the OA cartilage. Elevated expression of UCA1 promoted the chondrocytes cell proliferation and overexpression of miR-204-5p suppressed chondrocytes cell proliferation. In addition, overexpression of UCA1 decreased the expression of the type II collagen and type IV collagen expression in the chondrocytes. Elevated expression of miR-204-5p promoted the type II collagen and type IV collagen expression in the chondrocytes. We identified MMP-13 was a direct target gene of miR-204-5p in the chondrocytes. Overexpression of UCA1 enhanced the MMP-13 expression in the chondrocytes. Elevated expression of UCA1 regulated the chondrocytes cell proliferation and collagen expression through inhibiting the miR-204-5p expression. These results suggested that lncRNA UCA1 played as an important regulator of survival and matrix synthesis of chondrocytes partly through suppressing the miR-204-5p expression.

Previous studies demonstrated that lncRNA UCA1 played important roles in the development of tumors such as renal cell carcinoma, gastric cancer, colon cancer, hepatocellular carcinoma and osteosarcoma [34–38]. For example, Jiao et al. [39]. demonstrated that UCA1 expression was upregulated in the esophageal cancer tissues and ecoptic expression of UCA1 increased esophageal cancer cell proliferation by regulating the miR-204 and Sox4 expression. Chen et al. [40]. showed that UCA1 expression level was upregulated in the pancreatic cancer samples and the konckdown expression of UCA1 suppressed pancreatic cancer cell proliferation and increased the apoptotic rate and induced the cell cycle arrest. Lu et al. [41]. demonstrated that UCA1 expression level was higher in the lymph node metastasis samples than in the endometrial cancer tissues and the proliferative endometrium. Inhibition of UCA1 suppressed the endometrial cancer cell migration and invasion. However, the role of UCA1 in OA is still unknown. In this study, we found that the expression level of UCA1 was highest in the moderate and severe group compared to in the normal cartilage and mild OA cartilage. The data indicated that the expression level of UCA1 was upregulated in the OA cartilage. Elevated expression of UCA1 promoted the chondrocytes cell proliferation and overexpression of miR-204-5p suppressed chondrocytes cell proliferation. There results suggested that UCA1 play crucial roles in pathogenesis and progression of OA.

It has showed that lncRNAs act as regulators for miRNAs expression regulation. Previous studies showed that UCA1 promoted gemcitabine/cisplatin resistance by CREB regulating miR-196a-5p expression in the bladder cancer cells [28]. Fang et al. [34]. demonstrated that UCA1 iIncreased multi-drug resistance of gastric cancer through downregulating miR-27b expression. Bian et al. showed that UCA1 enhanced the 5-fluorouracil resistance and cell proliferation in the colorectal cancer through regulating the miR-204-5p expression. In line with this, we demonstrated that overexpression of UCA1 suppressed the miR-204-5p expression in the chondrocytes. The expression of miR-204-5p was downregulated in the OA cartilage. Moreover, the expression of miR-204-5p was negatively correlated with the UCA1 expression in the OA cartilage. Elevated expression of miR-204-5p promoted the type II collagen and type IV collagen expression in the chondrocytes. Moreover, we idetified MMP-13 was a direct target gene of miR-204-5p in the chondrocytes. Previous data demonstrated that MMP-13 was at low expression level in the articular cartilage during physiologic ECM turnover and was upregulated in human OA. MMP-13 can degrade the expressions of type 2 collagen and aggrecan. Furthermore, we showed that elevated expression of UCA1 regulated the chondrocytes cell proliferation and collagen expression through inhibiting the miR-204-5p expression.

In conclusion, we indicated that expression level of UCA1 was upregulated in the OA cartilage. Overexpression of UCA1 suppressed the miR-204-5p expression and enhanced the MMP-13 expression in the chondrocytes. These data provide the possibility of UCA1/miR-204-5p as therapeutic targets for the treatment of OA.

## MATERIALS AND METHODS

### Tissue samples

Normal knee cartilage samples were collected from patitents which were underwent the amputation due to trauma with no OA or rheumatoid arthritis history. Cartilage tissues of OA were obtained from OA patients who underwent knee replacement surgery. The diagnosed of these patients were followed to the criteria of American College of Rheumatology. This study was approved by Research Ethics Committee of the affiliated hospital of jining medical university and complied with Declaration of Helsinki. The written informed consent was collected from each participant. All cartilage tissues were divided into three groups following to the Kellgren/Lawrence Criterion: normal cartilage (K/L, Grade 0) for the normal group, low grade of OA cartilage (K/L Grade I and II) for the mild group, and high grade of OA cartilage (K/L, Grade III and IV) for the moderate and severe group.

### Cell culture and cell transfection

The human chondrocytes cell line C28/I2 (immortalized juvenile costal chondrocytes cell line) was purchased from ATCC (American Type Culture Collection, USA) and was cultured in the DMEM with fetal bovine serum (FBS), penicillin, and streptomycin. LncRNA UCA1 and control vector, miR-204-5p mimic and scramble were purchased from GenePharma (Shanghai, China). Cell transfection was performed by using the Lipofectamine 2000 (Invitrogen, USA) following to manufacturer’s information.

### Western blot analysis

Cell lysate was prepared by using the RIPA buffer and the protein concentration was measured by BCA protein kit (Pierce, Rockford, IL). Total protein was isolated through 12% SDS-PAGE and transferred to the nitrocellulose membrane. Membrane was blocked with 5 % nonfat milk and incubated overnight with the primary antibodies (MMP-13 and GAPDH, Sigma). After washing in TBST, the membrane was incubated HRP-conjugated secondary antibodies. The membrane was detected with ECL (enhanced chemiluminescence).

### RNA extraction and qRT-PCR

Total RNA was isolated by using the TRIzol reagent. Quantitative real-time PCR (qRT-PCR) was done by using SYBR GreenMix (Takara) on the MyiQ Real-Time PCR Detection System (Bio-Rad, CA). All the primers were shown as following: MMP-13, 5′-TGCTTCCTGATGACGATGTAC-3′, 5′-TCCTCGGAGACTGGTAATGG-3′; GAPDH, 5′-GACTCATGACCACAGTCCATGC-3′, 5′-AGAGGCAGGGATGATGTTCTG-3′. Relative gene expression level was mesured by using the ΔΔCT way and relative lncRNA expression was normalized to the U6 expression and mRNA expression level was normalized to GAPDH.

### Cell proliferation

Cells were cultured in the 96-well plate and continued to culture for 0, 24, 48 and 72 hours, respectively. Cell proliferation was measured by Cell Counting Kit 8 (CCK-8, Dojindo, Japan) following to the manufacturer’s information. The end product was determined spectrophotometrically at the wavelength of 450 nm.

### Luciferase reporter assay

The amplified DNA sequences were cloned to the pmiR-RB-REPORT™ Vector to form MMP-13 3′UTR (WT) and mutated MMP-133′UTR (Mut) luciferase vectors. For the reporter assay, cells were plated in the 96-well plate and were co-transfected with miR-204-5p mimic and scramble and MMP-13 3′UTR (WT) or mutated MMP-133′UTR (Mut) luciferase vectors. After 48 hours, the luciferase activity was determined by using Dual Luciferase Assay System (Promega, Madison, WI, USA) following to the manufacturer’s information.

### Statistical analysis

Value was shown as means ± (standard deviation) SD. The statistical assay was performed by using SPSS 17.0 (IBM Corporation, USA). Student t test was used to assess the significant difference between two groups and the differences between more than two groups were tested by one-way ANOVA. *P* < 0.05 was thought to be statistically significant.
